# Severe pneumonia combined with septic shock caused by community-acquired methicillin-resistant Staphylococcus aureus treated with veno-venous ECMO: A case report

**DOI:** 10.1097/MD.0000000000041627

**Published:** 2025-03-28

**Authors:** Linya He, Jian Wang, Yaxin Ning, Keqi Pan, Yan Chen, Jun Lu, Danqiong Wang

**Affiliations:** a The Second Clinical Medical College of Zhejiang Chinese Medical University, Hangzhou, Zhejiang, China; b The Third Clinical Medical College of Zhejiang Chinese Medical University, Hangzhou, Zhejiang, China; c Department of Critical Care Medicine, The Quzhou Affiliated Hospital of Wenzhou Medical University, Quzhou People’s Hospital, Quzhou, China; d School of Medicine, Shaoxing University, Shaoxing, China; e Department of Clinical Laboratory, Quzhou People’s Hospital, The Quzhou Affiliated Hospital of Wenzhou Medical University, Quzhou, China.

**Keywords:** acute respiratory distress syndrome, case report, community-acquired methicillin-resistant Staphylococcus aureus, linezolid, sepsis, severe pneumonia, veno-venous extracorporeal membrane oxygenation

## Abstract

**Rationale:**

Community-acquired methicillin-resistant *Staphylococcus aureus* (CA-MRSA) pneumonia is a severe and rapidly progressing infection that can lead to acute respiratory distress syndrome and septic shock. The use of venovenous extracorporeal membrane oxygenation (ECMO) may improve outcomes in critically ill patients who fail conventional mechanical ventilation.

**Patient concerns:**

Two female patients, aged 14 and 32 years, presented with fever and cough before hospital admission. Both patients rapidly developed severe respiratory distress and hemodynamic instability, raising concerns for a life-threatening infection.

**Diagnoses:**

Both patients were diagnosed with severe pneumonia caused by CA-MRSA, complicated by acute respiratory distress syndrome and septic shock. Microbiological testing confirmed the presence of CA-MRSA in respiratory samples.

**Interventions:**

The patients were initially treated with broad-spectrum anti-infective agents, including linezolid, targeting CA-MRSA. Due to the failure of conventional mechanical ventilation to maintain adequate oxygenation, venovenous ECMO was initiated to support respiratory function. The patients also received hemodynamic support and other adjunctive therapies for septic shock.

**Outcomes:**

Following the initiation of ECMO and targeted antibiotic therapy, both patients showed significant clinical improvement. Lung function recovered well, and they were successfully weaned off ECMO and mechanical ventilation. Both patients were eventually discharged with favorable outcomes.

**Lessons:**

CA-MRSA pneumonia can progress rapidly to severe respiratory failure and septic shock, necessitating aggressive interventions. Venovenous ECMO, combined with timely and appropriate antibiotic therapy can be life-saving in such cases. This report highlights the importance of early recognition, multidisciplinary management, and the potential benefits of ECMO in severe CA-MRSA pneumonia. It serves as a clinical reference for the treatment of similar cases.

## 1. Introduction

Methicillin-resistant Staphylococcus aureus (MRSA) is a term used to describe Staphylococcus aureus strains that contain the *mecA* gene or have an oxacillin MIC ≥ 2 μg/mL. These strains are resistant to β-lactam antibiotics^[1]^ and can cause various complex diseases including skin and soft tissue infections, necrotizing pneumonia, infective endocarditis, and bloodstream infections. The mortality rates of MRSA infections vary significantly depending on the infection site. The mortality from Staphylococcus aureus bacteremia is higher for methicillin-resistant Staphylococcus aureus (MRSA) than for methicillin-susceptible Staphylococcus aureus (MSSA), typically at 20% to 25%.^[2]^ MRSA pneumonia mortality to be around 30%, previous studies demonstrated that mortality was twice as high in patients with MRSA pneumonia than in patients with non-MRSA pneumonia. Additionally, community-acquired methicillin-resistant Staphylococcus aureus (CA-MRSA) infections generally have lower mortality rates (15–30%) compared to hospital-acquired MRSA (HA-MRSA) infections (30–50%), likely due to differences in patient comorbidities and bacterial resistance patterns.^[3]^ The diagnosis of purulent pericarditis from MRSA is extremely rare. However, the mortality rate for untreated patients can approach 100% but decreases to 40% in those patients who are appropriately treated with antibiotics and source control.^[4]^ In this report, we present 2 cases of teenage female patients who experienced severe pneumonia and severe acute respiratory distress syndrome (ARDS) due to CA-MRSA infection. The details of the conditions and treatments are described below.

## 2. Case 1

**Figure 1.**
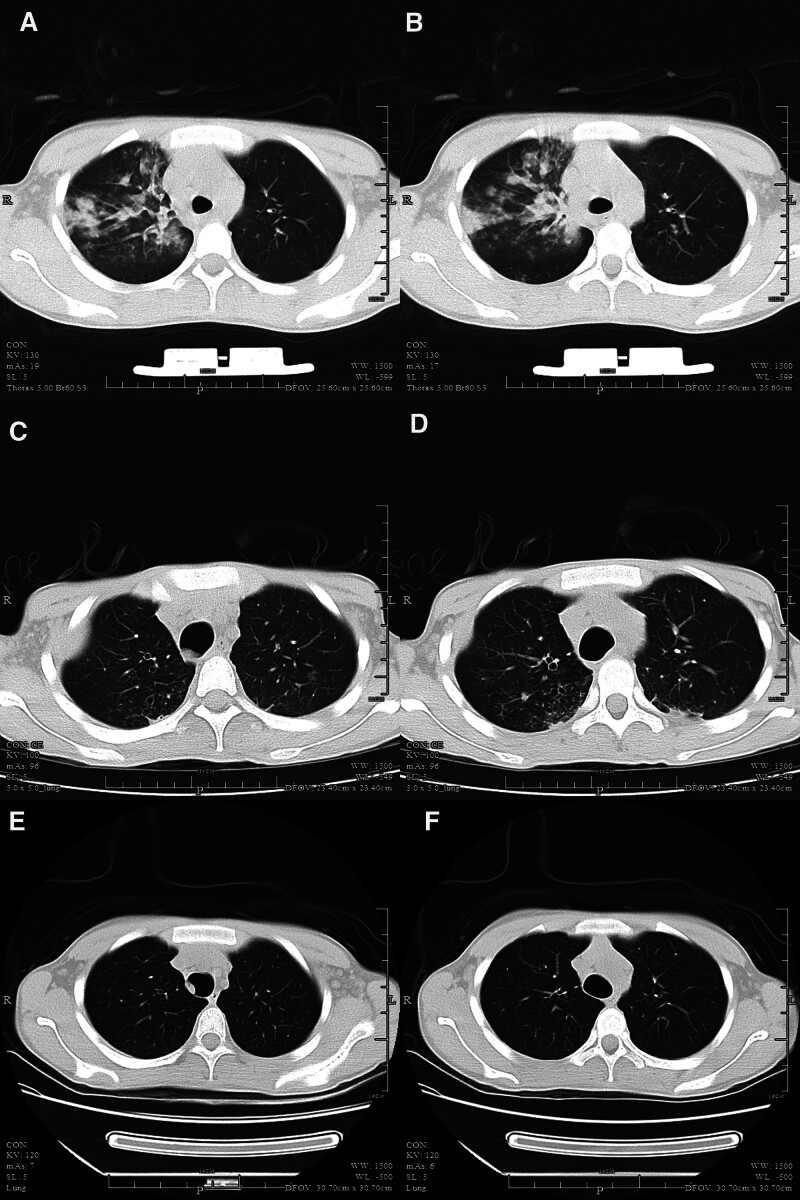
Imaging (A, B) February 24, 2022 chest CT revealed multiple infiltrations in both lungs. (C, D) April 25, 2022, chest CT showed less multiple infiltration in both lungs. (E, F) February 12, 2023 chest CT showed exudation lesions in both lungs accompanied by a few cystic shadows, which were smaller than before. CT = computed tomography.

A 14-year-old girl with a height of 156 cm and weight of 35 kg was admitted to the Infectious Disease Department of our hospital on February 24, 2022, with a cough accompanied by chest pain for 3 days and fever for 1 day. Upon admission, the patient presented with a body temperature of 38.2°C, pulse rate of 102 beats/minute, respiratory rate of 22 breaths/minute, blood pressure of 124/87 mm Hg, and blood oxygen saturation of 80% without oxygen inhalation. The patient was conscious and exhausted with slightly cyanotic lips, decreased breath sounds in the right lung, and cyanosis in the distal limbs. The results of the remaining physical examination were normal. Initial laboratory findings are shown in Table 1. Additionally, a chest CT scan showed multiple infiltrations in both the lungs (Fig. 1). The initial diagnosis was pulmonary infection.^[5]^ First, the patient received empiric anti-infective therapy, including intravenous ceftriaxone sodium (2 g) once daily and intravenous azithromycin 0.35 g once daily. Based on blood oxygen saturation and chest tightness, a mask oxygen inhalation was used to control symptoms. However, Despite oxygen therapy, the patient’s blood oxygen saturation only increased marginally from 80% to 81%, necessitating transfer to the intensive care unit (ICU) on February 24, 2022 for further treatment. Initial vital signs and arterial blood gas analysis results are shown in Table 1. Although conscious, the patient appeared mentally exhausted. Thick breath sounds and obvious rales were observed in both lungs, while the rest of the physical examination showed no remarkable findings. Based on the above results, The ICU diagnoses included community-acquired severe pneumonia, moderate ARDS, and septic shock.

**Table 1:** Clinical characteristics and laboratory findings of 2 cases on admission to ICU.

Parameters	Case 1	Case 2
Age (yr)	14	32
Initial symptoms	Cough with chest pain (3 d), fever (1 d)	Cough and sore throat (2 d), fever (0.5 d)
Vital signs at ICU admission		
*T* (°C)	38.0	39.1
HR (beats/min)	143	134
RESP (breaths/min)	25	34
BP (mm Hg)	152/73	134/65
SpO_2_* (%)	96	90
APACHE II	10	13
SOFA	6	6
Laboratory findings		
WBC (×10^9^/L)	6.6	9.9
NE (%)	88.0	85.6
LYM (%)	5.6	7.4
CRP (mg/L)	21.69	59.33
PCT (ng/mL)	3.48	7.77
pH	7.37	7.48
PaO_2_ (mm Hg)	120.0	68.6
PaCO_2_ (mm Hg)	38.6	31.2
HCO_3_^−^ (mmol/L)	22.1	22.9
Lac (mmol/L)	1.3	1.2
PaO_2_/FiO_2_	120.0	137.2

APACHE II = acute physiology and chronic health evaluation, BP = blood pressure, CRP = c-reactive protein, FiO_2_ = fraction of inspiration O_2_, HCO_3_^−^ = carbonic acid hydrogen radical, HR = heart rate, ICU = intensive care unit, Lac = lactic acid, LYM = lymphocytes, NE = neutrophils, PaCO_2_ = arterial carbon dioxide pressure, PaO_2_/FiO_2_ = oxygenation index, PaO_2_ = arterial oxygen pressure, PCT = procalcitonin, PH = hydrogen ion concentration, RESP = respiratory rate, SOFA = sequential organ failure assessment, SpO_2_ = arterial oxygen saturation, T = temperature, WBC = white blood cell.

* FiO_2_ = 10 L/min.

Tracheal intubation, fluid resuscitation, and ventilator-assisted breathing (pressure control 15 cm H_2_O positive end-expiratory pressure 10 cm H_2_O FiO_2_ 100%) were performed first. Based on the results of methicillin-resistant Staphylococcus aureus by culture of the bronchoalveolar lavage fluid specimen, follow-up treatment included intravenous infusion of linezolid 0.6 g every 12 hours, daptomycin 0.5 g once a day, and Piperacillin-Tazobactam 4.5 g every 8 hours. In addition, the patient received prone ventilation, continuous renal replacement therapy, and immunoadsorption therapy with oXiris filters.

**Figure 2.**
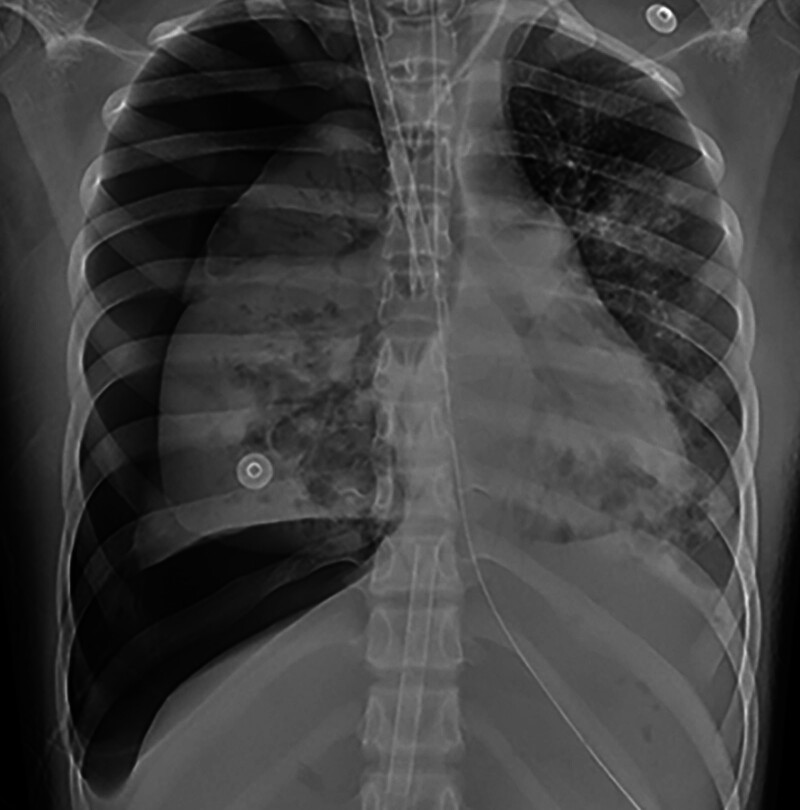
February 27, 2023 chest X-rays (CXRs) revealed right side liquid pneumothorax (gas about 60%). CXRs = chest X-rays.

**Figure 3.**
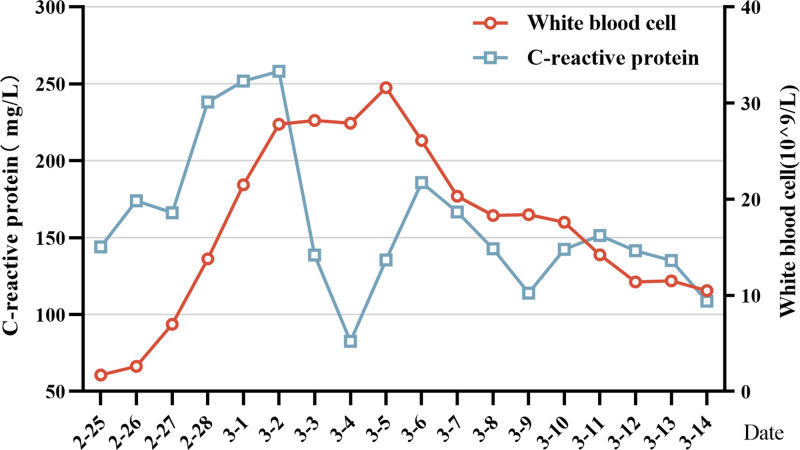
WBC count and CRP count decreased gradually during hospitalization. CRP = C-reactive protein, WBC = white blood cells.

On February 27, the patient experienced a drop in oxygen saturation to 84.6% and a decrease in blood pressure to 120/60 mm Hg. The patient was receiving norepinephrine at a rate of 0.91 µg/kg/minute and terlipressin at rate of 0.06 µg/kg/minute. A chest radiograph revealed a hydropneumothorax on the right side, with gas accounting for approximately 60% (Fig. 2). Despite thoracentesis and drainage, the patient’s oxygen saturation level did not improve, leading to initiation of VV extracorporeal membrane oxygenation (ECMO) treatment. The ECMO settings included a speed of 1900 rpm, flow rate of 2 L/minute, and gas flow rate of 1 L/minute for the air-oxygen stapler. During the hospital stay, the patient developed oliguria, which raised concerns regarding acute kidney injury. Therefore, oXiris immunoadsorption therapy was administered for 43 hours, followed by a switch to regular hemodialysis. On March 5, the patient’s blood parameters and inflammatory markers improved, and urine output returned to normal, leading to the discontinuation of CRRT. By March 10, the patient had regained consciousness and had stable circulation and oxygenation. A follow-up chest CT revealed a reduction in lung lesions compared to previous scans, and inflammatory markers such as white blood cells and C-reactive protein (CRP) were decreased (Fig. 3). Consequently, the patient was weaned off the VV-ECMO. On March 22, the patient experienced difficulty with extubation, resulting in tracheotomy. The tracheostomy cannula was removed on April 20 and the patient was transferred to the ICU on April 24. The patient was discharged on April 29. A 1-year follow-up chest CT scan showed an improvement in lung function (Fig. 1).

## 3. Case 2

**Figure 4.**
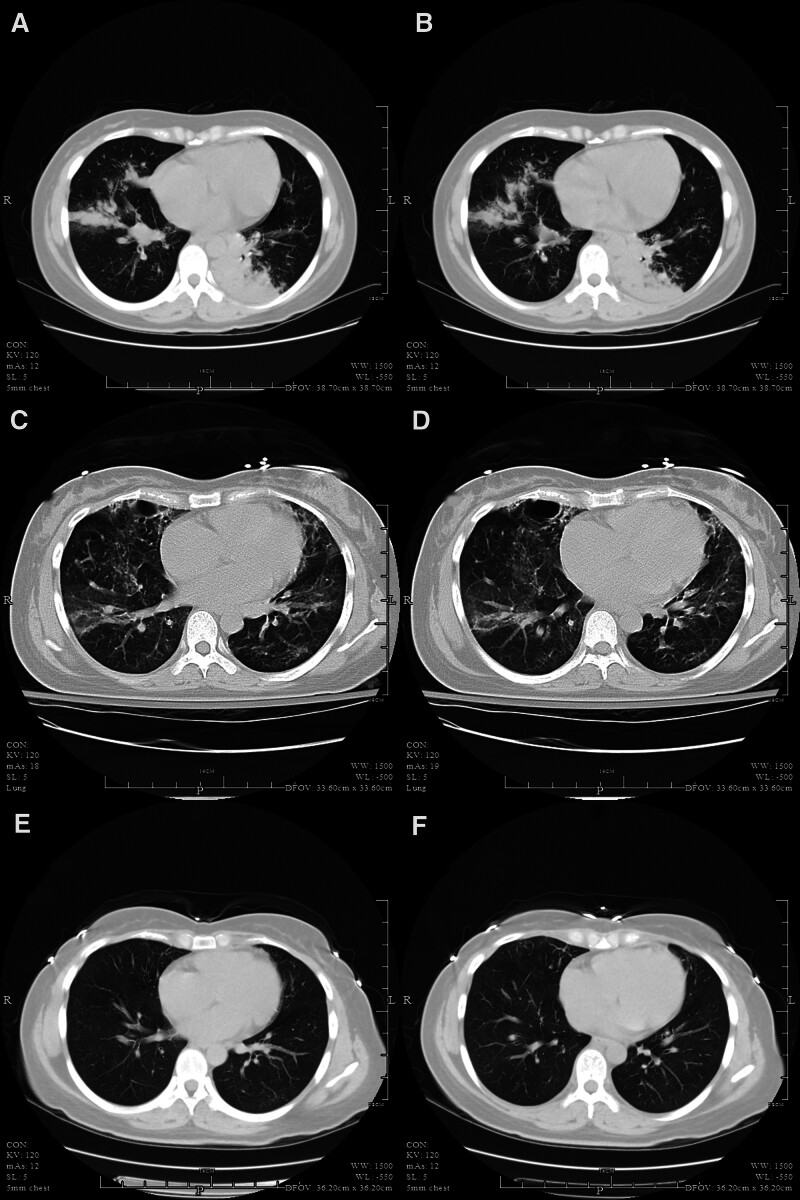
Imaging (A, B) March 3, 2023 chest CT showed multiple infiltrates in both lungs. (C, D) On April 2, 2023, chest CT showed slightly less multiple infiltration in both lungs. (E, F) July 29, 2023 chest CT showed multiple infiltrates in both lungs, which were fewer than before. CT = computed tomography.

A 32-year-old female with a height of 160 cm and weight of 61 kg was admitted to the obstetrics department of our hospital on March 2, 2023, due to cough and sore throat for the past 2 days, fever for half a day, and 20+1 weeks postmenopause. Upon admission, physical examination revealed a body temperature of 39.4°C, pulse rate of 107 beats/minute, respiration rate of 20 breaths/minute, blood pressure of 112/67 mm Hg, and SpO_2_ level of 95%. The patient was conscious and exhausted, with a red and swollen throat, but no suppuration. A lung auscultation revealed thick breath sounds and audible crackles. She had a history of cesarean section in 2020 and had been pregnant 3 times, giving birth to 1 child. The patient was allergic to penicillin and antibiotics. The uterine height was 18 cm, abdominal circumference was 85 cm, and fetal heart rate was 165 beats/minute. No obvious uterine contractions are observed. The results of the remaining physical examination were normal. Initial laboratory findings are shown in Table 1. Influenza A virus nucleic acid test results were positive. A chest CT scan conducted on March 3, 2023, revealed multiple infiltrates in both lungs (Fig. 4). The initial diagnosis was an acute lower respiratory tract infection, and empiric antibacterial treatment was initiated with intravenous administration of azithromycin 0.5 g once a day and oral administration of oseltamivir 75 mg once a day.

**Figure 5.**
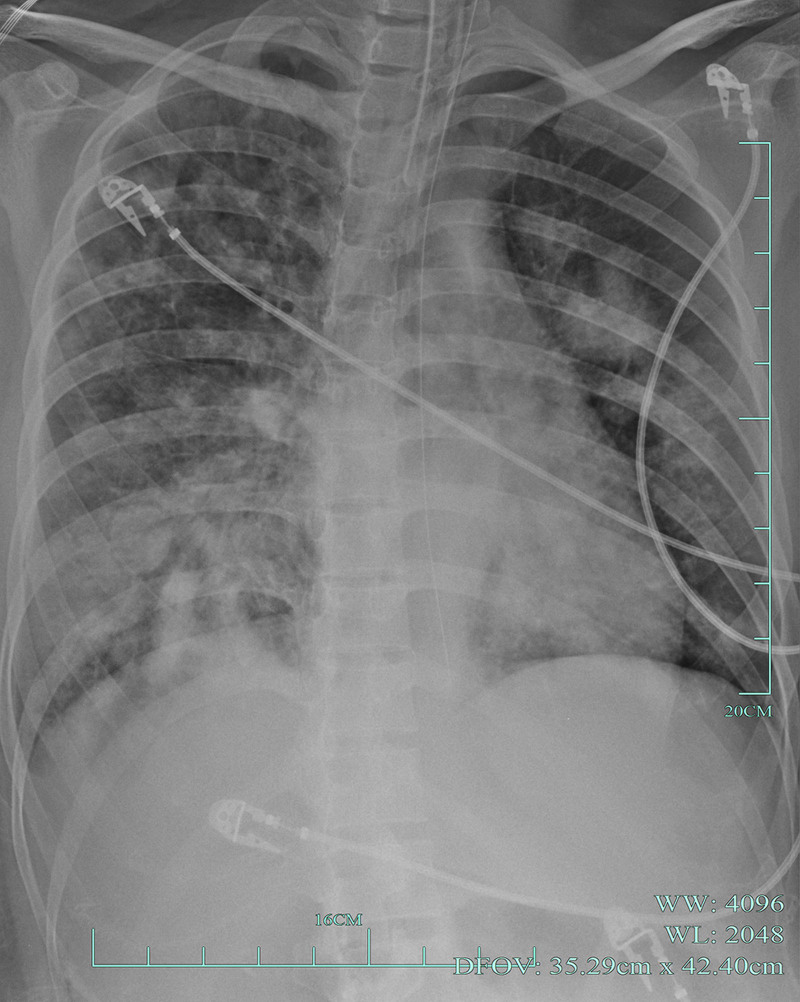
March 4, 2023 chest X-rays (CXRs) showed an increase in multiple infectious lesions in both lungs. CXRs = chest X-rays.

**Figure 6.**
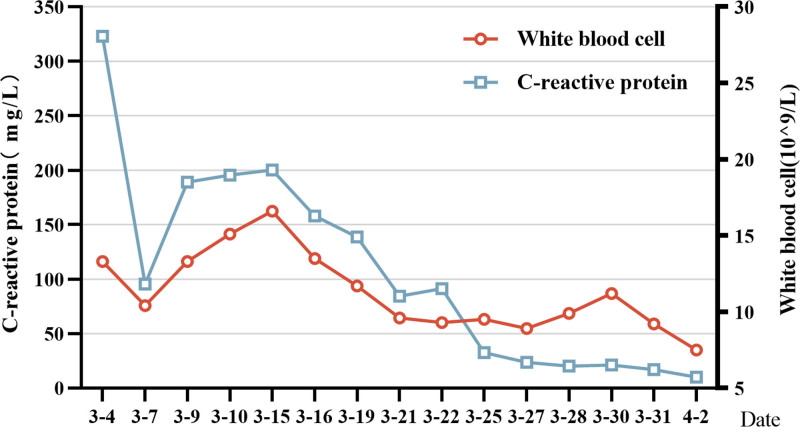
WBC count and CRP count decreased gradually during hospitalization. CRP = C-reactive protein, WBC = white blood cells.

After the patient was transferred to the ICU, her chest tightness and breathlessness worsened, and her blood oxygen saturation level was 89%. Initial vital signs and arterial blood gas analysis results are shown in Table 1. The initial diagnosis was severe viral pneumonia combined with moderate ARDS. The patient underwent tracheal intubation for mechanical ventilation. Fluid resuscitation was performed, and the patient received intravenous injections of imipenem 0.5 g and linezolid 0.6 g every 6 hours to prevent infection. Oseltamivir 75 mg was orally administered twice daily for antiviral treatment. On March 4, arterial blood gases analysis revealed a pH of 7.351, PaO_2_ of 68.0 mm Hg, PaCO_2_ of 37.4 mm Hg, SpO_2_ of 85%, fraction of inspiration O2 of 80%, and PaO2/FiO2 ratio of 85 mm Hg. A bedside chest radiograph showed an increase in multiple infectious lesions in both lungs compared with the previous images (Fig. 5). The patient was diagnosed with severe pneumonia and ARDS. Owing to difficulty in maintaining oxygenation, the patient underwent VV-ECMO. The ECMO settings were as follows: rotation speed 2500/minute, flow rate 3.5 L/minute, and air-oxygen stapler gas flow rate 3.0 L/minute. Methicillin-resistant Staphylococcus aureus was detected in the blood, alveolar lavage fluid, and sputum cultures on March 5. Based on the etiological examination results, the antibiotics were adjusted to daptomycin 0.5 g intravenously once a day, linezolid 0.6 g intravenously every 12 hours, and imipenem 0.5 g intravenously every 6 hours. On March 16, the levels of inflammatory indicators, such as white blood cells and CRP, decreased (Fig. 6). The patient’s bedside chest radiograph showed improvement in the lesions and respiratory conditions, leading to discontinuation of ECMO. A tracheotomy was performed on March 17, and the tracheostomy cannula was removed on April 4. The patient was transferred out of the ICU on April 5 and was successfully discharged from the hospital on April 14. A follow-up chest CT after 4 months showed improvement in lung function (Fig. 4).

## 4. Discussion

Studies have suggested that MRSA may be present in community-acquired infections.^[6]^ Blood culture results from both patients indicated the presence of CA-MRSA. This strain produces a leukocidal toxin with membrane-penetrating properties, leading to damage to the respiratory epithelial cells and cell lysis. Granulocytes release immune enzymes and cytokines to regulate inflammatory responses and decrease leukocyte count. CA-MRSA is highly pathogenic and can rapidly progress to necrotizing pneumonia and sepsis, posing a life-threatening risk of severe infections. The complexity of septic shock is intricate and may lead to multiple organ dysfunction syndromes. The central link between its occurrence and development is the cascade-like release of inflammatory factors.^[7]^ The OXiris blood filter has been demonstrated to be effective in removing cytokines and endotoxins. In comparison to conventional continuous veno-venous hemofiltration, using a blood filter with enhanced endotoxin/cytokine clearance in continuous veno-venous hemofiltration leads to a faster improvement in organ function within 48 hours.^[8]^ Various clinical studies have shown a notable decrease in the levels of inflammatory mediators and a reduced need for vasoactive drug doses in patients after treatment, resulting in significant improvements in hemodynamics.^[9–11]^

Linezolid, the first antibacterial agent approved by the oxazolidinone class, inhibits the synthesis of bacterial proteins and belongs to the class of bacteriostatic agents. It exhibits substantial antimicrobial activity against gram-positive organisms such as streptococci, staphylococci, and enterococci, including species that are resistant to conventional antibacterial treatment. Linezolid is fully bioavailable when administered orally compared to intravenous administration. Although higher concentrations of linezolid are observed in women than in men, this difference is not significant enough to require dosage adjustment.^[12]^ Vancomycin is currently the first-line drug for the treatment of methicillin-resistant Staphylococcus aureus (MRSA) infection, but it has been used in the clinic for a long time, has produced drug-resistant strains, its sensitivity to MRSA has decreased.^[13]^ Linezolid has a strong ability to penetrate the alveolar epithelial fluid and a higher clearance rate of MRSA compared to vancomycin, which makes it more effective in treating pneumonia.^[14,15]^ However, current research suggests that the combination of vancomycin and linezolid does not have a synergistic effect against MRSA strains, and may even result in an antagonistic effect.^[16]^ In addition to linezolid, clindamycin plays a crucial role in treating severe CA-MRSA infections. It acts as a protein synthesis inhibitor and has been shown to suppress toxin production in Staphylococcus aureus, particularly the Panton–Valentine leukocidin (PVL) toxin commonly associated with CA-MRSA strains. For severe cases of CA-MRSA pneumonia, combining clindamycin with other anti-MRSA antibiotics can potentially improve outcomes by reducing toxin-mediated tissue damage. Studies have demonstrated that early addition of clindamycin to standard therapy may reduce mortality in severe CA-MRSA infections, especially in cases presenting with toxic shock syndrome or necrotizing pneumonia. However, clinicians should be aware of the potential risk of inducible clindamycin resistance and perform D-zone testing before initiating therapy.

Mechanical ventilation is often used to provide respiratory support to ARDS patients. Despite the optimized ventilator settings, the 2 cases mentioned above still had oxygenation indices below 100 mm Hg; when mechanical ventilation was ineffective, VV-ECMO was a highly effective alternative.^[17]^ VV-ECMO helps remove carbon dioxide from the blood and improves oxygen transport by red blood cells, improving hypoxemia and buying time for further treatment of acute respiratory failure and acute respiratory distress syndrome. At the same time ECMO can prevent ventilator-related lung injury in a late stage.^[18]^ Early studies have shown that ECMO, particularly in patients with acute respiratory failure and acute respiratory distress syndrome, can improve survival rates.^[19–21]^ Two patients received ECMO early and survived severe hypoxemia, further reducing the systemic organ damage caused by severe hypoxia and preventing further exacerbation of the disease.

## 5. Conclusion

CA-MRSA infection can cause severe pneumonia with a rapid onset and serious prognosis, potentially leading to severe respiratory failure. However, with timely extracorporeal ECMO and active antibiotic therapy, patients have a good prognosis. This case report serves as a clinical reference for the treatment of severe pneumonia caused by CA-MRSA.

## Acknowledgments

The authors thank the patient and his family for allowing us to present the disease course in this report.

## Author contributions

**Data curation:** Linya He, Yaxin Ning, Keqi Pan, Jun Lu.

**Investigation:** Linya He.

**Methodology:** Linya He.

**Software:** Jian Wang.

**Supervision:** Danqiong Wang.

**Visualization:** Linya He.

**Writing – original draft:** Linya He.

**Writing – review & editing:** Linya He, Jian Wang, Yaxin Ning, Keqi Pan, Yan Chen, Danqiong Wang.
